# Efficient 3D Lidar Odometry Based on Planar Patches

**DOI:** 10.3390/s22186976

**Published:** 2022-09-15

**Authors:** Andres Galeote-Luque, Jose-Raul Ruiz-Sarmiento, Javier Gonzalez-Jimenez

**Affiliations:** Machine Perception and Intelligent Robotics Group (MAPIR-UMA), Malaga Institute for Mechatronics Engineering and Cyber-Physical Systems (IMECH.UMA), University of Malaga, 29071 Malaga, Spain

**Keywords:** mobile robots, localization and SLAM, 3D lidar, range sensing

## Abstract

In this paper we present a new way to compute the odometry of a 3D lidar in real-time. Due to the significant relation between these sensors and the rapidly increasing sector of autonomous vehicles, 3D lidars have improved in recent years, with modern models producing data in the form of range images. We take advantage of this ordered format to efficiently estimate the trajectory of the sensor as it moves in 3D space. The proposed method creates and leverages a flatness image in order to exploit the information found in flat surfaces of the scene. This allows for an efficient selection of planar patches from a first range image. Then, from a second image, keypoints related to said patches are extracted. This way, our proposal computes the ego-motion by imposing a coplanarity constraint between pairs <point, plane> whose correspondences are iteratively updated. The proposed algorithm is tested and compared with state-of-the-art ICP algorithms. Experiments show that our proposal, running on a single thread, can run 5× faster than a multi-threaded implementation of GICP, while providing a more accurate localization. A second version of the algorithm is also presented, which reduces the drift even further while needing less than half of the computation time of GICP. Both configurations of the algorithm run at frame rates common for most 3D lidars, 10 and 20 Hz on a standard CPU.

## 1. Introduction

For the safe navigation of autonomous vehicles it is essential to have an accurate estimation of their pose, that is, their position and orientation with respect to a given reference frame. These vehicles are typically equipped with sensors such as Inertial Measurement Units (IMUs), lidars or cameras, that allow them to perceive the vehicle and environment states and estimate their ego-motion. Regarding cameras, the most common approach for addressing this issue is *Visual Odometry* (VO), which consists of estimating the relative pose of the sensor along a sequence of images [1]. Similarly, *3D lidar odometry* permits the recovery of the vehicle motion along consecutive scans provided by a 3D lidar.

Particularly, 3D lidars stand out for yielding accurate geometric information from the scene in the form of point clouds, also known as *scans*, while performing reliably under changes in lighting conditions, which frequently occur in real-world scenarios [2]. The development of cheaper and more advanced 3D lidars has led to an increase in the popularity of these sensors for mobile platforms, especially autonomous vehicles. Examples of this are the autonomous driving platforms found in state-of-the-art projects such as Waymo [3,4] and Argoverse [5,6], as well as in datasets such as KITTI [7] or KAIST Urban [8].

The data points provided by traditional 3D lidars come in the form of unordered point clouds (i.e., a set of points defined by their Cartesian <X, Y, Z> coordinates, stored in an arbitrary manner). This brings 3D lidar odometry closer to the 3D registration problem: the issue of finding the rigid transformation between two consecutive point clouds, which, assuming a static world, corresponds to the sensor motion. In this regard, the Iterative Closest Point (ICP) algorithm [9], and its variants [10,11], are the most widespread methods mainly because they are effective and simple to implement. However popular, ICP algorithms are computationally expensive given the way the required calculations scale with the size of the point clouds. In a nutshell, ICP is based on finding, for every point in the source point cloud, the nearest point in the target point cloud, which requires calculating the distance between each possible pair of points. Thus, denser point clouds result in more distance calculations and, hence, a greater running time [12]. In order to obtain a correct convergence of the method, a high amount of points is needed, as the error yielded by each pair of points is averaged over the whole point cloud. This usually results in ICP running slower than the sensor frame rate, and therefore being forced to register a pair of non-consecutive scans with a greater relative motion. This becomes more of a limitation because of the serious chances of falling in local minima if the motion to recover is large, and/or no prior information about the motion is available for a good method initialization.

3D lidar sensors have seen an important improvement in recent years, with some modern models, especially those employing solid state technology, being able to produce an ordered point cloud, also referred to as ordered scan. These are composed of 3D points stored in a structured and consistent fashion. For example, the horizontal and vertical angles (polar and azimuth, respectively) of the spherical representation of the points can be equally distributed along the field of view. This enables the ordered scan to be represented as a range image, where the row and column relate to the azimuth (θ) and polar (ϕ) angles, respectively, and the value of each pixel represents the distance to the sensor. Having access to the geometric information of the scene with this new format paves the way for the development of novel odometry methods, which can capitalize on ordered point clouds to achieve a higher efficiency. In addition to this, nowadays the move of autonomous vehicles towards optimizing Size, Weight and Power (SWaP) [13] is clear, resulting in systems with scarce computing resources available to share between the different processes running on the platform. As a consequence, the usage of fast and efficient algorithms becomes a strong requirement.

**Figure 1:**
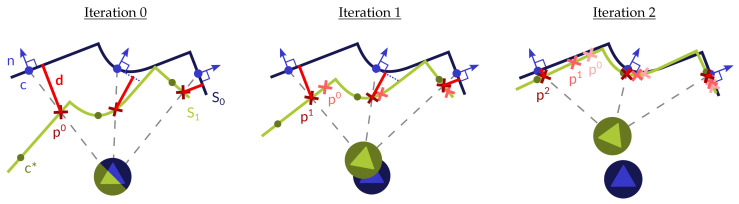
Consecutive iterations of the proposed algorithm. First, certain areas of the ordered scan $S_{0}$ (dark blue line) are selected based on their flatness. Planar patches defined by their center (c, blue dot) and normal (n, blue arrow) are fitted to the selection. These centers are projected (gray dashed lines) based on the current motion estimation onto $S_{1}$ (light green line), where the point ($p^{k}$, red cross) related to said plane can be extracted (with *k* representing the iteration). By imposing the coplanarity constraint [14], the relative pose of the sensor can be calculated as the rigid transformation that minimizes the point-to-plane distances (*d*, red line) between each pair <point, plane>. In the next iteration, the estimated motion is used to update the point to which each plane is matched. This improves the probability of each pair fulfilling the coplanarity constraint, and thus results in a more accurate estimation of the movement. Notice how the selected point becomes closer and closer to its corresponding centroid ($c^{*}$, dark green dots) with each iteration.

In this paper we propose an efficient 3D lidar odometry method that performs at a high frame rate by taking advantage of the structure of ordered scans provided by modern 3D lidars. These data come in the form of range images, allowing us to leverage techniques popular in traditional Visual Odometry, especially in depth VO, which tend to be more efficient than ICP-like 3D registration methods. In the proposed method, the motion is recovered by imposing the coplanarity constraint [14] between pairs <point, plane> extracted from the input images. This reduces to finding the rigid transformation that minimizes the point-to-plane distance between pairs of features, similar to *indirect/feature-based* VO methods [15,16]. However, we skip solving for correspondences (e.g., using a minimum distance criterion) since the matches are iteratively established based on the projection function of the sensor, similar to what is adopted in *direct* methods such as [17,18]. The result is a hybrid algorithm that combines techniques from both *direct* and *indirect* VO methods to obtain the odometry of a 3D lidar by exploiting the flatness of surfaces present in most scenes, typically in man-made environments, both indoors and outdoors. Because we are imposing the coplanarity constraint to pairs <point, plane>, we are assuming that each point comes from its counterpart planar patch. This holds when the planar patches are sufficiently flat to be approximated by a plane, and their supporting regions are large enough with respect to the sensor relative pose. Thus, selecting planar patches that satisfy these requirements is essential in our method, and occupies much of the computation time. Also notice that, as the method approaches convergence, the motion to recover becomes smaller, making it more probable for the coplanarity constraint to be fulfilled. With each iteration of the algorithm, each point slides along their corresponding planar patch towards its centroid. In an ideal case, the points becomes coincidental with the centroid of their matched planar patch at convergence. Figure 1 shows a representation of the proposed technique applied to a simple scene, with consecutive iterations of the algorithm being shown.

It is worth mentioning that the applicability of the method could be extended to any sensor that delivers any kind of point cloud, such as traditional 3D lidar and even depth cameras, by creating a range image representation through the projection of the point cloud onto a spherical surface.

To validate the presented method, we have compared different configurations of it with state-of-the-art techniques over the dataset presented in VelodyneSLAM [19]. The results show how our method is capable of running in a single thread at real-time (∼30 Hz), five times faster than a multi-threaded GICP competitor, also outperforming the accuracy of other methods at a fraction of the computational cost. An alternative multi-threaded configuration has been also studied, reporting a higher accuracy while halving the computation time of GICP.

## 2. Related Work

For the purpose of clarity this section will be divided into two subsections: *Depth Visual Odometry* and *3D Lidar Odometry*. The proposed method leverages techniques found in depth VO, and in order to contextualize said techniques the state of the depth VO literature will be presented first. We will then proceed to review the state of the art regarding 3D lidar odometry, to correctly establish the place of the proposed method in the literature.

### 2.1. Depth Visual Odometry

Depth visual odometry applied to RGB-D cameras has been broadly researched since the appearance of these sensors. The approaches found in the literature can be divided into two main categories: *indirect* and *direct* methods. The former, also known as *feature-based*, are characterized for reducing the input images to a set of features which are matched by solving correspondences between them. The motion of the sensor is estimated by minimizing the distance between pairs of features. Examples of features studied in the literature are points [20], a combination of points and lines [21], and planes [15,16]. The correct finding of correspondences among features is essential in indirect methods; hence, most of the computation time is spent in this step. In particular for planar features, a plane segmentation algorithm is usually applied, which tends to slow the system down due to it being time-consuming.

On the contrary, direct methods avoid extracting features and instead minimize an energy function that is usually related to the difference between consecutive depth and/or RGB images when one of them is reprojected back against the other. Steinbrücker et al. [22] presented this concept applied to both RGB and depth images, where the photometric error is minimized, while the only purpose of the geometric information is to reproject one image against the other. This work was extended in [17] by introducing a new probabilistic formulation that produced better results. The work presented by Jaimez and Gonzalez-Jimenez [18] only utilizes the geometric information found in the depth image, estimating the motion by applying the range flow constraint densely. The result is a very fast and accurate depth odometry method, outperforming similar approaches found in the literature at the time.

Although not an odometry method, the Iterative Closest Point [9] has served as the comparison baseline to most of the depth odometry approaches. ICP was formulated for the registration of two unordered point clouds. Since depth images are a representation of an ordered point cloud, ICP can be applied to obtain the relative pose of the sensor given a pair of frames. Different versions of the ICP algorithm have been developed through the years: in [10] the point-to-plane distance is minimized instead of the original point-to-point; Generalized ICP [11] combines the two previous methods into a single probabilistic framework. The same idea was then expanded by Servos et al. [12] by adding more information in the form of channels, such as color or intensity, to reduce the ambiguity of the solution.

The method proposed in this paper could be categorized as *indirect* because it makes use of features (points and planes). As explained before, solving for correspondences is an essential procedure in traditional *feature-based* methods, because having features matched incorrectly usually results in a detriment in accuracy. We circumvent this critical step by iteratively matching the features based on the current relative pose, as in *direct* methods, thus allowing for more leniency in the pairing of the features and a faster execution time.

### 2.2. 3D Lidar Odometry

Despite 3D lidars producing geometric information too, such as RGB-D cameras, the unordered point clouds provided by early technology could not be represented as an image, rendering the previously mentioned VO methods unusable. The localization problem related to 3D lidar was seen closer to 3D registration, and thus the ICP algorithm and its variations dominated the literature. Nonetheless, over the years, other approaches have been developed, such as the 3D Normals Distributions Transform (NDT) [23], which stands out for offering an alternative to ICP. NDT was originally conceived for 2D localization [24], where the model is represented by a combination of normal distributions, describing the probability of finding a surface point at a certain position. Similar to the previously mentioned indirect methods, the proposal of Velas et al. [25] matches line features to estimate the odometry of a 3D lidar sensor.

One of the most common applications of 3D lidar odometry is as the front-end of SLAM systems, and even though it has not been as studied as depth odometry, the literature displays a wide collection of 3D lidar SLAM algorithms. In spite of the fact that there are SLAM methods where a custom feature-based odometry has been implemented [26,27], most of the 3D lidar SLAM systems perform the scan-to-scan alignment with some variation of ICP (see [19,28,29,30,31]).

In recent years, 3D lidars have been developed to produce ordered point clouds which can be represented as a range image, specially with the introduction of solid state lidar. This new representation of the geometric information of the scene fosters the creation of new methods that exploit its advantages. Given the novelty of these 3D lidars capable of generating ordered scans, the literature does not yet reflect this trend. In our proposal, we leverage the image representation of the ordered point clouds by employing techniques found in depth VO. This allows it to be a fast 3D lidar odometry method, which is desirable for various reasons. First, as the front-end of a SLAM system, having the localization available at a higher frame rate means the SLAM algorithm has more information to work with, while leaving more computational resources free for other processes running alongside. Additionally, the development of fast odometry methods suits the advance in robotics and autonomous vehicles towards optimizing Size, Weight and Power (SWaP).

## 3. Method Overview

**Figure 2:**
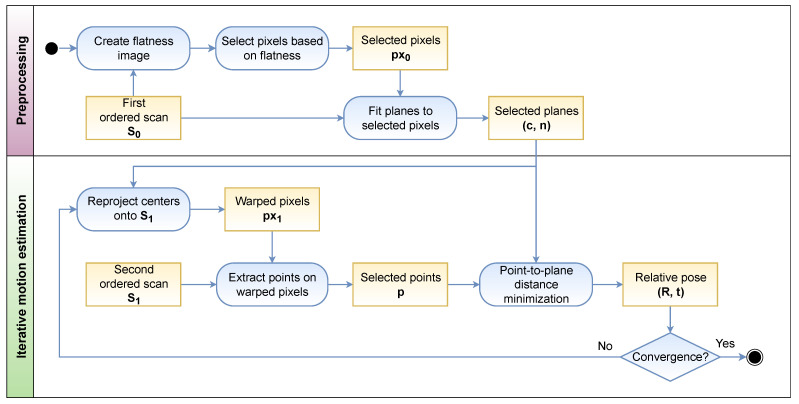
Overview graph showing the workflow of the proposed method. As a preprocessing step, a flatness image is computed from the first ordered scan $S_{0}$ to find the pixels most likely located in flat surfaces of the scene. Once planes have been fit to the selected pixels, the iterative motion estimation process can begin. It consists of reprojecting the selected planes onto $S_{1}$ based on the current motion estimation, which yields their corresponding pixel coordinates. From these image coordinates, the point related to the selected plane can be extracted, completing the feature pair <point, plane>. The relative pose estimation is then obtained by minimizing the point-to-plane distance of feature pairs. This is repeated until convergence.

This section summarizes how the proposed method estimates the motion of a 3D lidar over a sequence of range images. Thus, the objective is to find the relative pose of the sensor between two consecutive frames, defined by the rotation matrix and translation vector (R,t), with R∈SO(3) and $t\in R^{3}$. The input is a pair of ordered scans, represented as range images, $S_{0}$ and $S_{1}$, where $S_{i}:\left(\Omega \in N^{2}\right)\to R$ is defined on the image domain Ω. Figure 2 shows the general workflow of our method.

The main idea of this method is to create pairs of planar and point features from $S_{0}$ and $S_{1}$ respectively, to then impose the coplanarity constraint to each pair. When this hypothesis holds true, the rigid transformation that minimizes the point-to-plane distance between them corresponds to the sensor motion [14]. Note that the supporting flat surface from which the planar patch is extracted does not need to be completely flat for the coplanarity constraint to apply. The bigger the supporting flat surface of the planar features, the higher the probability of fulfilling the hypothesis. Thus, a selection algorithm is employed to choose the pixels more probable of belonging to large flat surfaces.

**Figure 3:**
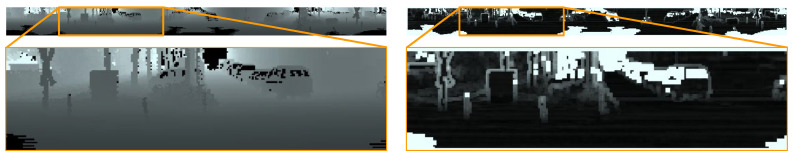
Flatness image (**right**) generated from $S_{0}$ (**left**) applying the LSF method. Flat surfaces such as the ground and walls appear darker, while non-planar pixels are displayed brighter. Images below are zoomed in areas of the panoramic images above.

The approach followed in the proposed algorithm consists of creating a flatness image (as seen in Figure 3) that holds the information of how flat the neighborhood around each point is, so pixels can be selected based on this score. Once the pixels from $S_{0}$ located in flat surfaces have been selected, a planar patch is fitted to the neighborhood of each one. The details of this selection process will be further explained in Section 3.1.

The next step consists of finding the 3D points from $S_{1}$ related to each selected plane. This is an iterative process, since the points are extracted from the pixel coordinates obtained by reprojecting the planes onto $S_{1}$ based on the current motion estimation. For the first estimation, the movement is assumed to be zero, resulting in matching each planar feature with the 3D point in $S_{1}$ located in the same pixel coordinates. This will be expanded on Section 3.2.

This reprojection-based matching results in a set of planes from $S_{0}$ defined by their center and normal vector $\left(c_{i},n_{i}\right)$, and their corresponding points $p_{i}$ from $S_{1}$, with $c_{i}\in C$, $n_{i}\in N$ and $p_{i}\in P$. The relative pose (R,t) that minimizes the distance between the pairs of features also represents the motion between the input scans. It is possible, as long as the coplanarity constraint is fulfilled and each point belongs to the corresponding plane, to register both scans this way. Section 3.3 will delve into this estimation process.

As explained before, this is an iterative process, so the estimated motion is fed back to recalculate the points associated to each plane. This increases the probability of the paired features fulfilling the coplanarity constraint, which in turn improves the estimation of the relative pose. This can be repeated until convergence.

### 3.1. Flatness-Based Selection

This section describes the preprocessing step which reduces the input frame $S_{0}$ to a set of selected planar patches defined by their centers *C* and normal vectors *N*. These planar patches will be, at a later stage, matched with points *P* from $S_{1}$. This pairing will be considered valid if both features belong to the same surface of the scene, satisfying the coplanarity constraint. The fulfillment of this hypothesis depends on two main factors: the size of the selected planar patches and the movement to recover. Note that the latter is given by the speed of the sensor and cannot be controlled, unlike the former. To increase the probability of planar features being correctly matched, they are selected based on how flat their neighborhoods are. The bigger the supporting region of the selected plane ($c_{i}$,$n_{i}$), the higher the probability for the point $p_{i}$ to lie within it in the next frame, even after big movements. Some form of plane segmentation could be applied here to improve the selection, but these algorithms tend to be computationally expensive, preventing the method from running at real time. However, as previously discussed, opting for a faster alternative has the advantages of working with smaller movements, having the solution (pose estimation) available more often.

Considering this, the proposed selection approach is based on obtaining a flatness image, which holds information about how planar the area around each pixel of $S_{0}$ is. An example of a flatness image is shown in Figure 3. In this article, two ways of obtaining the flatness images are presented, each one prioritizing accuracy or efficiency, respectively:

- **Least Squares Fitting (LSF)**: For each pixel, a plane is fitted to its neighborhood using LSF. Then, the error between the fit plane and the surrounding points is used as the value in the flatness image. This technique is very accurate at the cost of being computationally more expensive.
- **Curvature-based (Fast method)**: This approach exploits the function obtained by representing a 3D plane in the spherical coordinates of the range image. These images describe each point in spherical coordinates, where the value of each pixel is the distance (*r*) from the point to the sensor, and the image row and column relate to the azimuthal (θ) and polar (ϕ) angles, respectively. When a simple plane such as (1) is represented in said spherical coordinates system, the distance *r* of a point in the plane can be described as a function of ϕ and θ as in (2).
  (1) ax+by+cz=1
  (2) $$r\left(\phi ,\theta \right)=\frac{1}{a\cos\phi \cos\theta +b\sin\phi \cos\theta +c\sin\theta }$$
  Thus, the inverse of the distance function is a sine wave (3).
  (3) $$d\left(\phi ,\theta \right)=\frac{1}{r}=a\cos\phi \cos\theta +b\sin\phi \cos\theta +c\sin\theta$$
  Instead of trying to fit a sine wave to check for flat areas, the curvature of the neighborhood can be tested. The curvature of a function can be approximated by its second derivative [32], which means that the second derivative of the inverse of the distance *d* along both ϕ and θ should be low for pixels located in flat surfaces. However, non-planar areas, such as the intersection of two planes, will result in an abrupt change in curvature. The derivatives along both image axes can be efficiently calculated with the use of 2D convolutions, applying the Sobel operator. The value stored in the flatness image is the curvature (*k*) of each pixel, calculated as the sum of the absolute value of the second derivative of *d* along each axis (4).
  (4) $$k=|\frac{\partial^{2}d}{\partial \phi^{2}}\left|+\right|\frac{\partial^{2}d}{\partial \theta^{2}}|$$

Regardless of the technique used to obtain the flatness image, a Gaussian filter is applied to blur it afterwards, which helps to deal with pixels located near non-planar areas by leveraging the score of their neighborhoods.

Once the flatness image has been created, it is then used to select pixels from $S_{0}$ based on their score. To ensure a good distribution of pixels around the scene, the image is divided into blocks, and the best pixels from each block are selected. This also serves as a non-maximum suppression step.

A planar patch ($c_{i}$, $n_{i}$) is fitted to each neighborhood around the selected pixels in the image $S_{0}$, with the centroid of the group being the center of the plane. For the normal vector, the singular value decomposition is applied to the matrix created by stacking the difference of each point of the neighborhood and the centroid. The normal vector is then the left singular vector corresponding to the least singular value. A measurement of the fitness $f_{i}$, how planar the group of points is, is also estimated as the least singular value. Low values of this variable mean the group of points actually form a planar surface.

Take into account that it is no guarantee that the selected pixels are located in a planar surface just from the flatness image, but once the planes are fit into their neighborhood, the fitness $f_{i}$ can be used to downweight selected pixels found near occlusions or in curved surfaces (see Section 3.3).

### 3.2. Iterative Projection-Based Matching

After the planar patches have been obtained from $S_{0}$, their corresponding points $p_{i}$ have to be extracted from $S_{1}$. In traditional indirect methods, the features from both frames are matched based on some descriptor. Instead, in the proposed method, an iterative approach is implemented, inspired by direct methods. It consists of reprojecting the centers of the selected planes onto $S_{1}$ based on the current motion estimation, to find the pixels in $S_{1}$ that are more probable of representing the same scene surface as their corresponding planar region. This process works as follows:

1. The center of each plane $c_{i}$ is transformed using the motion estimation (R,t) to obtain $c_{i}^{*}$:
  (5) $$c_{i}^{*}={\left[x_{c},y_{c},z_{c}\right]}^{T}=R^{-1}\left(c_{i}-t\right)$$
2. The spherical coordinates of the transformed center are then calculated:
  (6) $$\left\{\begin{array}{cc} r_{i} & =\sqrt{x_{c}^{2}+y_{c}^{2}+z_{c}^{2}}\\ \phi_{i} & =\arctan2(y_{c},x_{c})\\ \theta_{i} & =\arcsin(z_{c}/r_{i}) \end{array}\right.$$
3. The row (*v*) and column (*u*) on the image are obtained based on the spherical coordinates:
  (7) $$\left\{\begin{array}{c} v_{i}=\arg\min_{j}\left|\theta_{vec}\left(j\right)-\theta_{i}\right|\\ u_{i}=\left[\left(\mathrm{columns}-1\right)\left(\pi -\phi_{i}\right)/\left(2\pi \right)\right] \end{array}\right.$$
  where $\theta_{vec}$ represents the vector of the azimuthal angles related to the row numbers, and [] is the rounding operator, which returns the integer number closest to the input value.
4. These pixel coordinates can then be used to obtain the points $p_{i}$ from $S_{1}$. A Gaussian filter is applied over the neighboring points to reduce negative effect of noise.

During the first iteration, the relative pose between both range images is considered to be composed by the identity rotation R=I and no translation t=0, which means that the points $p_{i}$ are located in the same image coordinates as the centers $c_{i}$ of the selected planes, but in the next image. Note that by imposing the coplanarity constraint the only requisite is that the points and planes selected belong to the same surface of the 3D scene, so there is no need for a perfect point-to-point matching. By selecting a pixel in $S_{0}$ located in a planar surface as explained in the previous section, there is a high probability that the point located at the same image coordinates in both frames lies on the same surface.

### 3.3. Relative Pose Estimation

This section describes how the relative pose between the two range images is calculated given the set of matching <point, plane> pairs, assuming they satisfy the coplanarity constraint. The objective is to find the rigid transformation (R,t) that minimizes the point-to-plane distance of each pair of features, as shown in Equation (8). This is optimized by the Levenberg–Marquardt implementation from the well-known Ceres Solver [33]:

(8) $$\left(R,t\right)=\arg\min_{R,t}\sum_{i}\rho \left({∥\alpha_{i}n_{i}\cdot \left(Rp_{i}+t-c_{i}\right)∥}^{2}\right)$$

where $\alpha_{i}=\left(1-f_{i}\right)$ serves as a weight of the residual based on the fitness of the selected plane. This way, selected pixels located in surfaces with lower flatness have less impact on the final result. The Huber loss function ρ is applied to the residual to deal with outliers, which are pixels that do not fulfill the hypotheses, whether because they move with respect to the static scene or because they do not lie in the same surface in both frames. Along with the relative pose, the covariance of the solution is calculated, which holds the information of the uncertainty of each parameter of the rigid transformation.

The motion estimation is part of the iterative process previously mentioned (recall Figure 2), so the resulting transformation is fed back to update the points *P* in $S_{1}$ corresponding to each planar region. This improves the matching of the features by making it more probable that both of them belong to the same surface of the scene, improving in turn the motion estimation. This procedure is repeated until the convergence of the solution.

As a final process, a motion filter is applied to the resulting relative pose, similar to the one found in [18], in which the motion estimation from the previous pair of frames is leveraged if the covariance of the current solution is high. This has proven to be helpful in scenes with low planar information, improving the accuracy of the localization as will be proven in Section 4.4.

## 4. Experiments and Results

In this section, we evaluate the performance of our method resorting to sequences of 3D lidar scans from the widely-used Velodyne SLAM dataset [19] (see Section 4.1). The obtained results are compared with different implementations of the state-of-the-art GICP algorithm [11], including single-thread and multi-thread alternatives (Section 4.2 and Section 4.3). For all the experiments, the test platform used is a standard PC with 8GB of RAM, running Ubuntu 20.04 with an Intel Core i7-7700HQ CPU with four cores and eight execution threads at 2.8 GHz. We also present an ablation study exploring different configurations of the proposed method (Section 4.4) and discuss the limitations of the proposal (see Section 4.5).

### 4.1. Testbed

The proposed method was evaluated on the two outdoor sequences provided by the Velodyne SLAM dataset [19], captured by a vehicle driving around town in a closed loop over a kilometer in length. The Velodyne HDL-64E sensor, running at 10 Hz, provides the geometric information in two formats: point clouds and 870 × 64 range images.

The error metric employed to compare the different methods is the one proposed in [34], known as relative pose error (RPE). Although the RMSE of the error will be shown because of its popularity in the odometry literature, the mean RPE will be used to compare the methods based on the arguments presented in [35].

The proposed method is configured with the following parameters:

- Neighborhoods are 3 × 3 blocks of pixels.
- The flatness image is divided into 16 × 16 blocks, and the four best pixels are selected from each block.

### 4.2. Time Performance

The performance of our method is compared to the widely popular GICP algorithm [11] as implemented in two different C++ libraries: PCL [36] and Open3D (O3D) [37]. The former is a basic single-thread (ST) implementation, while the latter applies a loss function to reduce the effect of outliers, and performs at a higher rate because of multi-threading.

The proposed method is tested with both versions of the flatness image introduced in Section 3.1, *fast* and *LSF*, with the second method being more accurate at the cost of computation time. For a fair comparison with the GICP implementation of Open3D, a multi-threaded (MT) implementation of the LSF variant is included. A breakdown of the elapsed times for every part of the proposed algorithm is shown in Table 1 for both versions. It is clear how the use of 2D convolutions in the *fast* version of the method greatly reduces the computational cost. Regarding the threaded implementation of our method, the only process worth parallelizing is the creation of the flatness image, resulting in a 59% reduction of computation time.

**Figure 4:**
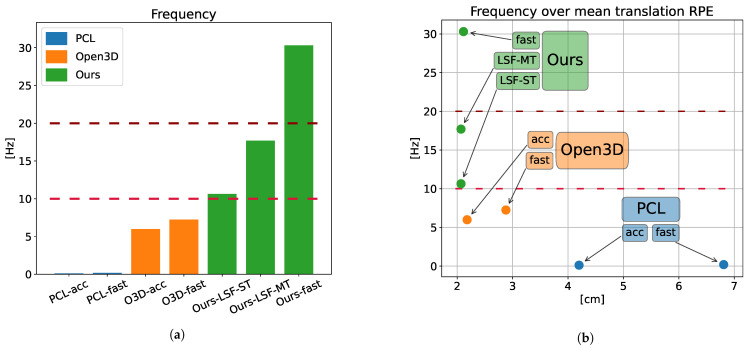
Frequency comparison of the different methods. (**a**) Frame rate of the different methods. The usual working frequencies of 3D lidar, 10 and 20 Hz, are displayed along the data. (**b**) Frequency of the different methods over the translational RPE. The usual working frequencies of 3D lidar, 10 and 20 Hz, are displayed along the data.

With the goal of performing a balanced comparison, a pair of configurations were employed in the GICP calculations, each one prioritizing accuracy or speed over the other one. In summary, seven odometry methods are compared in this section, four of them being a variation of GICP (PCL-fast, PCL-acc., Open3D-fast, Open3D-acc.), and three of them being the different versions of the proposed method (Ours-fast, Ours-LSF-ST, Ours-LSF-MT). The Open3D implementation of GICP and Ours-LSF-MT are multi-threaded, while the rest run on a single thread. Table 2 shows the computation time and frequency of each method, where we can see how the slower implementation of our method, using the LSF, outmatches the frame rate of the faster GICP (Open3D-fast), while the fast version achieves 4× its speed. Note that 3D lidar sensors usually run at 10–20 Hz, so the presented configurations of the proposed method work in real time. Figure 4a shows this information in a more visual way.

### 4.3. Accuracy Results

**Figure 5:**
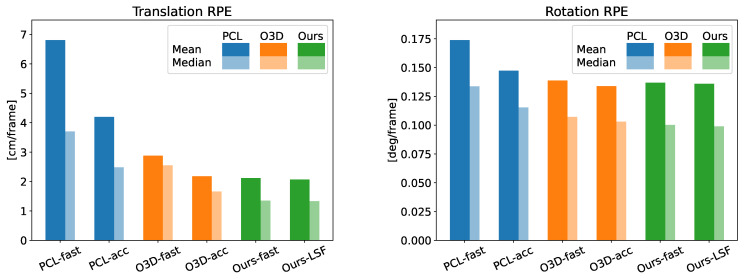
Comparison of the mean and median RPE of the different methods. Translational and rotational error are displayed on the left and right, respectively.

**Figure 6:**
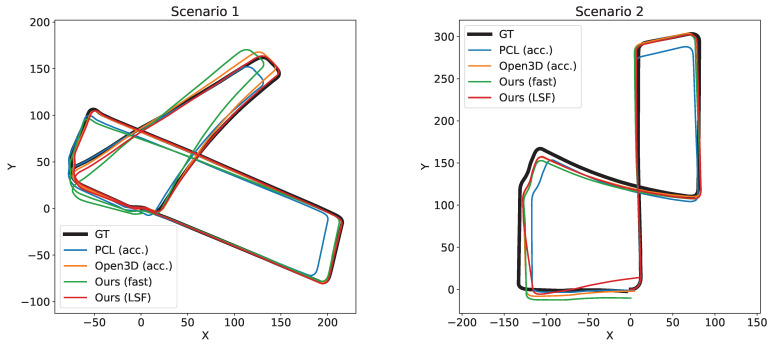
Top view of the resulting trajectories of the tested methods over each sequence of the dataset, along with the ground truth.

Regarding the accuracy, Table 3 displays the translational and rotational error of the six methods being compared along the two sequences of the dataset. Note that the results obtained from Ours-LSF are the same whether or not it is multi-threaded. In said table we can see how, when comparing the LSF version of our method with the competitor showing the lowest error (Open3D-acc), our proposal is able to achieve a reduction in the translational error of 5% and 19% of the mean and median value of the RPE, respectively, while speeding up its frame rate by a factor of 2.9×. Even the proposed Ours-fast version, which works five times faster than Open3D-acc., reaches a similar accuracy improvement. Regarding the rotation error, a similar performance is achieved, with the difference between GICP and our method being below 3%. Note that there is a notable difference between comparing the RMSE, mean error, and median error of the different methods. We agree with [35] that the RMSE is not able to correctly capture the general accuracy of the data since it severely penalizes instances of big errors, however scarce they are. The mean absolute error on the other hand allows for a fairer comparison of the accuracy of the presented methods. In addition, the median absolute error conveys the accuracy obtained in most of the frames along each sequence. Figure 5 shows a visual representation of this evaluation, where we can check the superior performance of our proposal regarding accuracy. Moreover, for a qualitative comparison, the trajectories computed by each method are displayed in Figure 6.

Figure 4b shows the running frequency of each method over their mean translation RPE, allowing a comparison of accuracy and speed combined. For example, it can be seen how Ours-fast only performs 3% better than O3D-acc in translation RPE, but runs 5× faster. Compared to the faster version of GICP, Ours-fast works at 4x the speed, and the accuracy improvement goes up to 26%. Regarding the *LSF* version of the proposed method, it yields a better accuracy, with a 5% and 28% improvement over the accurate and fast versions of Open3D, respectively, while running 2.9× and 2.4× faster than them.

### 4.4. Ablation Study

The purpose of this section is to validate the contribution of the different processes employed in our proposal to the resulting performance. Particularly, three procedures are analyzed: the iterative update of the correspondences, the motion filter (see Section 3.3), and the blurring of the flatness image (Section 3.1). Thus, the baseline for this test is the base algorithm running a single iteration.

Based on Table 4, the effect of a second iteration proves to be worth it, reducing the translation error over 70%. Allowing the system to iterate until convergence improves the accuracy even further. Regarding the motion filter, it helps to mitigate the effect of scenes where not enough information is found (see Section 4.5), which happens sparsely throughout the sequences, resulting in an improvement of the accuracy of 3%. Similarly, applying a Gaussian filter to the flatness image helps with selecting pixels located far from non-planar areas, reducing the error 2.6% further.

### 4.5. Limitations

The formulation of the presented method entails the limitation caused by the aperture problem [38], meaning that certain scenarios exist in which the motion cannot be estimated caused by the lack of planes oriented in different directions. A clear example of these undetermined scenes would be a corridor, in which the translation along it cannot be recovered by imposing the coplanarity constraint. To solve this issue, in addition to the motion filter, information from another sensor, like IMUs or RGB cameras, could be leveraged to constrain the solution.

## 5. Conclusions

In this paper we have introduced a novel method for 3D lidar odometry that exploits the structure of the range images that modern sensors provide, as well as information from flat surfaces to achieve run-time operation and a low drift. These flat surfaces are leveraged to extract planar patches, described by their centroid and normal vector, from the first range image, which in turn are used to retrieve features in the second image in the form of points. The utilization of a flatness image is proposed to efficiently select planar patches, and two different ways to estimate it have been presented, each one prioritizing speed or accuracy over the other. The coplanarity constraint is imposed to each <point, plane> pair. This way the motion is estimated by minimizing the point-to-plane distance between pairs of features. Their correspondences are updated iteratively based on the current estimation of the relative pose, by employing the projection model of the sensor. When the point related to each plane is updated based on the current pose estimation, the coplanarity constraint becomes more probable to be fulfilled, thus improving the accuracy of the solution. Gaussian and motion filters are used to reduce the negative influence of noise and scenarios with limited information, respectively.

To test its viability, our approach has been compared with GICP, widely used in 3D lidar SLAM systems. Two GICP configurations have been used in the comparison, one focusing on accuracy and the other on speed, to match the two versions of the proposed method. When comparing the fast version of both approaches, our method turns out to be capable of achieving four times the frame rate using a single thread, while improving the accuracy by 26%. Regarding the implementations where the goal is to maximize the accuracy, our approach runs 2.95× faster than GICP, and yields 5% more accuracy. This reduction in computation time allows the resources of the system to be shared between the different processes running alongside.

In future works, the adaptability of the method parameters will be studied. For example, the size of the neighborhood can change depending on the distance to the point, or quadtrees can be used instead of fixed-size blocks in the selection. Being adaptive to the peculiarities of the scene at hand would permit the method to properly operate with a wider range of real-world conditions.

## Figures and Tables

**Table 1:** Computation time breakdown of the proposed method.

Process	Fast (ST)	LSF (ST)	LSF (MT)
Flatness image	2.5 ms	63.5 ms	26 ms
Plane select. and fitting	3ms	3ms	3ms
Matching points extraction	2.5 ms	2.5 ms	2.5 ms
Motion estimation	25 ms	25 ms	25 ms
**Total**	33 ms	94 ms	56.5 ms

**Table 2:** Computation time comparison between the different methods.

Threaded	Method	Time per Frame	Frame Rate
No	PCL (fast)	5300 ms	0.188 Hz
	PCL (acc.)	8800 ms	0.114 Hz
	Ours (fast)	33 ms	30.30 Hz
	Ours (LSF, ST)	94 ms	10.64 Hz
Yes	Open3D (fast)	138 ms	7.24 Hz
	Open3D (acc.)	167 ms	5.98 Hz
	Ours (LSF, MT)	56.5 ms	17.69 Hz

**Table 3:** RPE comparison between the different methods.

(a) Translational RPE.							
[cm/frame]		PCL	PCL	O3D	O3D	Ours	Ours
		fast	acc.	fast	acc.	fast	LSF
RMSE	Scen. 1	11.55	8.54	5.68	**5.14**	6.95	7.04
	Scen. 2	14.68	9.22	**7.47**	7.54	7.98	7.83
	Avg	13.12	8.88	6.58	**6.34**	7.47	7.44
Mean	Scen. 1	6.55	4.31	2.97	2.30	2.22	**2.18**
	Scen. 2	7.07	4.09	2.79	2.06	2.01	**1.96**
	Avg	6.81	4.20	2.88	2.18	2.12	**2.07**
Median	Scen. 1	3.79	2.58	2.31	1.77	1.47	**1.44**
	Scen. 2	3.61	2.38	2.79	1.55	**1.23**	**1.23**
	Avg	3.70	2.48	2.55	1.66	1.35	**1.34**
**(b) Rotational RPE.**							
[deg/frame]		PCL	PCL	O3D	O3D	Ours	Ours
		fast	acc.	fast	acc.	fast	LSF
RMSE	Scen. 1	0.230	0.198	0.189	**0.184**	0.212	0.203
	Scen. 2	0.228	0.177	0.168	**0.163**	0.257	0.324
	Avg	0.229	0.188	0.179	**0.174**	0.235	0.263
Mean	Scen. 1	0.177	0.154	0.147	**0.142**	0.145	0.144
	Scen. 2	0.171	0.140	0.131	**0.126**	0.128	0.129
	Avg	0.174	0.147	0.139	**0.134**	0.137	0.136
Median	Scen. 1	0.134	0.119	0.112	0.109	0.108	**0.107**
	Scen. 2	0.133	0.111	0.102	0.097	0.093	**0.091**
	Avg	0.134	0.115	0.107	0.103	0.100	**0.099**

**Table 4:** Improvements in accuracy of the different versions of the proposed method.

Version	Translation: Mean RPE (cm/frame)	Improvement over Previous Version
Base (1 iteration)	11.27	-
Base (2 iterations)	3.24	71.25%
Iterations until convergence	2.31	28.70%
Prev. and motion filter	2.24	3.03%
Prev. and flatness image blurring	2.18	2.68 %

## Data Availability

Not applicable.

## Abbreviations

The following abbreviations are used in this manuscript:

ICP	Iterative Closest Point
GICP	Generalized Iterative Closest Point
VO	Visual Odometry
SWaP	Size, Weight and Power
NDT	Normals Distribution Transform
SLAM	Simultaneous Localization and Mapping
LIDAR	Light Detection and Ranging
LSF	Least Squares Fitting
RPE	Relative Pose Error
RMSE	Root Mean Square Error
ST	Single Thread
MT	Multi-Threaded
IMU	Inertial Measurement Unit
